# Supplementary material for: Secondary motor integration as a final arbiter in sensorimotor decision-making
Source: PLoS Biol. 2023 Jul 17;21(7):e3002200. doi: 10.1371/journal.pbio.3002200 (PMC10393169; doi:10.1371/journal.pbio.3002200)
Supplement: S1 Text — S1 Table A. Constraints of model parameters across conditions, for each of the models under consideration (see Fig 2A–2D). The constraints are given per model (row): A dot indicates that this parameter is fixed across all conditions; S indicates that the parameter may change between stimulus conditions; and U indicates that the parameter may change between urgency conditions. S1 Table B. Prediction simulation parameters used for Fig 3. (DOCX) [file pbio.3002200.s001.docx]

**Supporting information**

| **Model** | $\boldsymbol{T}_{\boldsymbol{er}}$ **distribution** | $\boldsymbol{a}$ | $\boldsymbol{x}_{\boldsymbol{0}}$ | $\boldsymbol{v}_{\boldsymbol{i}}$ | $\boldsymbol{\lambda}$ | $\boldsymbol{c}$ |
| --- | --- | --- | --- | --- | --- | --- |
| LIT | U | . | . | S | U | . |
| Fixed boundary | U | U | . | S | . | . |
| Collapsing boundary | U | . | . | S | . | U |

***Table A. Constraints of model parameters across conditions****, for each of the models under consideration (see Fig 2A-D). The constraints are given per model (row): a dot indicates that this parameter is fixed across all conditions, S indicates that the parameter may change between stimulus conditions and U indicates that the parameter may change between urgency conditions.*

| **Figure** | $\boldsymbol{a}$ | $\boldsymbol{\lambda}^{\boldsymbol{-1}}$ | $\boldsymbol{v}_{\boldsymbol{1/2}}$  **(weak)** | $\boldsymbol{v}_{\boldsymbol{3/4}}$  **(strong)** |
| --- | --- | --- | --- | --- |
| 3A | 1 | 0 | 0.5 | 4 |
| 3B | 0.5 | 0.1 | 0.5 | 4 |
| 3C | 0.5 | 0.1 | 0.5 | 4 |
|  |  | 0.2 |  |  |
|  |  | 0.3 |  |  |

***Table B.*** *Prediction simulation parameters used for Fig 3.*
