# Supplementary material for: Secondary motor integration as a final arbiter in sensorimotor decision-making
Source: PLoS Biol. 2023 Jul 17;21(7):e3002200. doi: 10.1371/journal.pbio.3002200 (PMC10393169; doi:10.1371/journal.pbio.3002200)
Supplement: Attachment — Submitted filename: ResponseToReviewers.docx [file pbio.3002200.s002.docx]

*We thank the reviewers for their careful consideration of this manuscript and their constructive comments. These comments have been incredibly helpful for improving the clarity of the manuscript and ensuring the rigor of the findings, thus making the manuscript more accessible to a wider audience.*

*To ensure we have thoroughly addressed the reviewer’s comments, and to simplify the identification of changes in the manuscript we have added an identification code to each comment in the format RxCy, where x corresponds to the reviewer number and y corresponds to the comment number. Where necessary, we have split the comments into parts a and b. An example: R2C3b corresponds to reviewer 2 comment 3 part b. We provide a supplementary document where the changes in the manuscript are highlighted and commented with the corresponding identification code. To help reviewers find the changes, we refer to the page number and line number with PxLy: P2L103 refers to page 2 line 103.*

Reviewer #1:

This review was prepared by Jeff Schall. I am familiar with and have benefitted intellectually from the authors' previous D*M work and the Leaky Integrating Threshold (LIT) model. I am very sympathetic to the authors' theoretical stance in this work because, as they know, it aligns so well with numerous observations we and others have made, and it duplicates theoretical stances we have adopted previously. Here, the authors have used their insightful and original LIT model to distinguish EEG signals putatively associated with stimulus categorization and response preparation. The approach is sophisticated. The results are not beyond methodological critique by skeptics, but they are intelligible and entirely consistent with previous observations. The interpretation follows naturally from the results. The manuscript is written clearly enough for diverse scientists. The theoretical perspective and the observations should make a very useful contribution to the literature on model-based decision making of general interest to a broad audience. However, as detailed below, the manuscript can be improved in scholarship to enhance its impact.

*We are grateful for the detailed feedback from Dr Schall, whose expertise continues to be especially valuable for improving the manuscript. We hope to have thoroughly addressed the comments below.*

**R1C1**
(1) The authors' appeal to theoretical novelty in the Introduction and Discussion seeks to reach unnecessarily far. In the Introduction, the authors write, "These recent ﬁndings suggest that neural activity related to motor preparation, starts its buildup before the sensory evidence integration completes, effectively lagging the primary process of evidence accumulation. Consequently, the amount of sensory evidence could have a direct impact on motor planning, which in turn could inﬂuence the eventual choice. This entanglement is not accounted for in current incarnations of decision models nor has it been characterized at the level of neuronal responses." This summary and the conclusion are factually incorrect. Multiple neurophysiological studies have described motor preparation beginning before sensory evidence integration completes. Also, the claim that no "incarnation of decision models" accounts for this is contradicted by the "Gated Accumulator Model" formulated by Tom Palmeri, Gordon Logan, and me with our trainees. Now, in Discussion, the authors do acknowledge the alignment of the LIT structure with numerous preceding neurophysiological investigation. They are careful to distinguish LIT as a "formal, fully estimatable model", and that is so. But the authors could be more generous in acknowledging other model-based frameworks that anticipate their original approach such as the "Gated Accumulator Model" (Purcell et al 2010; 2012). Yes, they cite one of the original publications, but the text in the Introduction obscures the convergence of outlooks, which reinforces their preferred theoretical stance. Also, the text in the Discussion offers a naïve reader no insight into the conceptual relationships between these two bodies of research. Such an explanation would not detract from but would rather amplify the authors' impressive demonstration.

*We thank Dr Schall for highlighting the lack of clarity in this paragraph in the introduction, and how it can be better linked with the discussion. We have changed the paragraph in the introduction to the following:*

*“Multiple neurophysiological studies have described activity related to motor preparation beginning before sensory evidence integration completes, effectively lagging the primary process of evidence accumulation. This would allow the amount of sensory evidence to have a direct impact on motor planning, which is not accounted for in traditional evidence accumulation models. While some recent models have captured the build-up of motor preparation signals using inhibitory- (“Gated Accumulator Model”; Purcell et al., 2010; 2012 [25, 26]) or urgency- (“Urgency Gating Model”; Cisek et al., 2009; Thura et al., 2012; Thura et al., 2014 [27 – 29]) gating, they do not preserve a separate sensory evidence accumulation signal, and thus do not explicitly capture the entanglement of sensory evidence accumulation with motor preparation”* ***P1L33***

*As Dr Schall suggests, we have attempted to give the naïve reader greater insight into the conceptual relationships between these two bodies of research in the discussion. We have edited the following sentence:*

*“The smoothing of the evidence accumulation process happening at the LIT stage makes the effective noise of the final accumulator auto correlated, which is a fundamental difference from most choice RT models, including other leaky integration and gating models with evidence filtering57–60.”*

*To:*

*“The smoothing of the evidence accumulation process happening at the LIT stage makes the effective noise of the final accumulator auto correlated, which is a fundamental difference from most choice-RT models: In previous models with evidence gating, the evidence becomes effectively amplified (for urgency-gating; Cisek et al., 2009; Thura et al., 2012; Thura et al., 2014 [27-29]), or selectively inhibited (in the case of the gated accumulator model; Purcell et al., 2010; 2012 [25,26]), but there is no temporal smoothing. Without temporal smoothing, changes in the amplification/inhibition of evidence over time can be computationally approximated by changes in the temporal profile of the decision boundary (Drugowitsch et al., 2012 [59]). In contrast, double integration provides clearly distinguishable computational predictions promoted by the temporal lag involved in smoothing.”* ***P16L469***

**R1C2**
(2) The authors' treatment of the recent literature on neural mechanisms of SAT can be improved substantially. They conclude that "SAT is likely controlled by changes in the proposed leaky motor integration process rather than the long-standing view of boundary adjustments". This conclusion is anticipated by but does not acknowledge as clearly as it might our original observations about the neurophysiology of SAT (Heitz & Schall 2012; see also Reppert et al. 2018). The authors also write, "These findings also help reconcile recent neural and computational reports showing little evidence of collapsing decision boundaries during SAT manipulations.", citing Heitz and Schall 2012 #49 and Hanks et al. 2014 #50 among others. Unfortunately, this sentence conveys only the least of what we and others have found. We discovered that the neurons identified with evidence accumulation and response preparation in frontal eye field and in the superior colliculus did not vary in 'threshold' as predicted by the standard accumulator models (reviewed by Bogacz et al 2010 #32 in the manuscript). The authors may not have noticed, though, that we also discovered that SAT instructions also profoundly affected the neurons representing the evidence for the decision in this task. When we fed these neural data into the Gated Accumulator Model (Servant et al. 2019 - which they now cite), it captured key measures of performance, but flexible combinations of gate and threshold parameters were necessary to explain differences in SAT strategy across monkeys. Does LIT have enough parameters to do this?

*We agree with Dr Schall’s assertion that the issue of the neural mechanisms of SAT is rather complex. While the Bogacz review is a very helpful reference for the broader audience, a more recent review would be helpful for providing the level of details and significant advances that support our manuscript. However, we are not aware of a more recent review. We have tried to better articulate the points above while also balancing the need to be concise.*

*We hope that the additional issue of the change in the accumulated evidence signal is sufficiently introduced via the change in the introductory paragraph in response to comment 1 above. We have additionally alluded back to this in the discussion:*

*“These findings also help reconcile recent neural and computational reports showing little evidence of collapsing decision boundaries as the primary control mechanism for SAT manipulations (Heitz et al., 2012; Hanks et al., 2014; Voss et al., 2019; Rafiei & Rahnev, 2021; Reppert et al., 2018 [51-55]) but rather, highlight the involvement of changes in the acceleration of evidence toward the bound (Servant et al., 2019 [56]).”* ***P15L446***

*And weakened the statement from the introduction, while at the same time emphasizing the contribution of animal electrophysiology studies that have also offered evidence against the traditional view on boundary adjustments:*

*“In doing so, we validate the newly proposed framework and simultaneously demonstrate that the SAT is likely primarily controlled by changes in the proposed leaky motor integration process rather than the long-standing view of boundary adjustments [8,35]. Our findings help reconcile the emerging inconsistencies in explaining SAT, most notably from animal electrophysiology experiments (Heitz & Schall, 2012; Reppert et al., 2018 [36,37]), and provide a foundation upon which future studies can continue to interrogate the neural systems underlying rapid sensorimotor decision-making.”* ***P2L52***

*We agree that it is possible that additional parameters may be necessary to fully capture other data sets. It is certainly possible to implement both changes in leak and bound for modulating SAT in the LIT, which should provide the same flexibility as the combination of changes in gate and threshold parameters in the gated accumulator model. However, in this manuscript we focussed on the key differences between the double integration framework and traditional evidence accumulation frameworks, most importantly, the leak. We show that modulating the leak provides a better description of SAT control than boundary adjustments, highlighting the importance of this process. Our findings are in line with the statement that changes in leaky motor integration* ***primarily*** *control SAT, since we show that this is a better description of the data than changes in the bound.*

**R1C3**
(3) In citing the publications addressing collapsing bounds, the authors do not distinguish the claims and positions of authors on both sides of the question. Some authors would be unhappy to be listed alongside others as if this is a decided question or that it is applicable under all testing conditions. The authors can correct this easily by rephrasing sentences and separating citations.

*We thank Dr Schall for pointing this out, we have adjusted the sentence as follows:*

*“An emerging trend in the literature focuses on the potential utility of single-accumulator models with collapsing boundaries [33,34; Palestro et al., 2018; Glickman et al., 2022] (though this remains contentious [31,32] Hawkins et al., 2015; Voskuilen et al., 2016).”* ***P15L430***

The authors may also wish to consider these additional comments:

**R1C4**
[A] In Discussion, the authors state that they "…show computationally that the LIT … offers a better fit to behavior compared to traditional single accumulator diffusion models…". The authors should explain how this claim relates to other publications that contrast quantities like BIC. For example, is it not the case that this approach will not scale with either the number of parameters or the number of models?

*We thank Dr Schall for this comment, which highlighted to us how our modelling approach may not be so accessible to a wider audience. For this reason, we additionally fit the models using the more common approach of minimising the negative loglikelihood over RT-quantiles estimated using simulation. With this approach, the LIT has an additional parameter and so BIC is the more appropriate metric for model comparison. Our conclusions with this approach remain the same: the LIT provides a better computational description of the data. Including this additional analysis allowed us to clarify the model comparison of the D*M approach, as well as provide BIC which relates better to other publications (BIC cannot be straight-forwardly computed for the D*M criterion). First, we emphasise that in the reparameterization of the LIT into DDM parameters, all models have the same number of parameters:*

*“We emphasise that changes in the inverse leak were reparameterised as equivalent changes in the DDM parameters, such that all of the resulting models have the same number of free parameters, and we can use their fit to the data as a way to arbitrate between the mechanisms (Fig 2A-D).”* ***P5L149***

*We then contrast this approach with the simulation-based approach:*

*“In addition, we fit a standard DDM and the LIT (without reparameterising) using the more common simulation-based approach to estimate the negative log-likelihood of response-time quantiles (see* ***Materials and Methods****). The predicted choice-RT distributions of these models are quite similar (****Fig 2E****). In this case the LIT has an additional parameter compared to the DDM and yet not only showed superior negative log-likelihood (*$\sum{NLL}_{LIT}={6.430e}^{4}; \sum{NLL}_{DDM}={6.448e}^{4}$*) but also survived the penalty for the additional parameter afforded by the Bayesian Information Criterion (*$\sum{BIC}_{LIT}={1.311e}^{5}; \sum{NLL}_{DDM}={1.312e}^{5}$*; on average, LIT BIC was 1.38 less than the BIC for the DDM ). Thus, these two model fitting approaches suggest similar conclusions: that the leak mechanism of the LIT provides a better computational description of SAT control in the behavioural data.”* ***P5L155***

**R1C5**
[B] The analysis pipeline for the EEG data included several choices that a skeptic could query. The manuscript would be improved if the authors demonstrated the robustness of the observations relative to variation of the several steps and parameters used in the analysis.

*We agree with Dr Schall that the EEG analysis is novel, which may draw skepticism from some readers. There are two issues with this: First, without established standards and testing across more datasets, it is unclear whether the somewhat arbitrary choices with parameters (such as the duration of the time-windows) and metrics (such as the choice of summary statistic) will affect the results; and second, whether the novel analysis methods are written with sufficient detail to repeat the analysis (and thus assess the first issue). In addressing these issues, we show that our results are robust to variation in several key steps of the analysis.*

*We address the second issue by bringing in a colleague (Tarryn Balsdon) whose expertise overlaps with this work, to re-run the analysis, writing scripts from scratch, based solely on what is written in the manuscript. We have added details to the methods where the exact analysis was unclear:*

1. *The normalisation of the evidence accumulation signals* ***P24L686***
2. *The window over which the moving average was implemented prior to selecting the signal-RT zero crossings* ***P24L687***
3. *The use of an intercept term in calculating the regression coefficient for the signal slope snapshots* ***P24L701***

*While repeating the analysis, Tarryn chose different metrics:*

1. *z-scoring the accumulation signals before averaging (example 1 below),*
2. *calculating the signal-RT correlations using the log reaction times (so that the reaction times are roughly normally distributed; example 3 below)*
3. *using a fisher transform on correlation coefficients prior to averaging across participants, and*
4. *calculating parametric between-subject confidence intervals.*

*We switch to these in the new manuscript, as these metrics are more in line with standard statistical recommendations. Further, we assess the impact of several somewhat arbitrary parameter choices: the choice of time-window duration and the selection of critical time-points.*

*We present plots of these variations in the analysis steps below, but in the manuscript we briefly mention how these parameters can be manipulated without affecting the results:*

*“The EEG-predicted evidence accumulation signals were computed using the spatial filter (w) from the window around the response (t = 0; though choosing a window prior to the response produces similar results)”* ***P24L684,*** *example 2 below*

*“Each slope snapshot is calculated based on the raw signals of a specific trial and pertains to individual 50 ms time windows within that trial (decreasing this duration gives similar, though more noisy results).”* ***P24L700*** *example 5 below*

*“For each trial, we calculate a 50 ms window slope snapshot every 25 ms with window centres ranging from 150 ms to 50 ms before the response (though the window centres can be extended further prior to the response without affecting the results).”* ***P24L703*** *example 6 below*

*We believe this process has addressed both of the issues with the novelty of the analysis as fully as we can with the current dataset. We will additionally include that analysis scripts with the data, so that the skeptic can easily manipulate the analysis steps if they have concerns, and so others can easily re-use this analysis methodology. We thank Dr Schall for the suggestion, which has strengthened our confidence in the robustness of the results.*

*Here we show several variations in analysis steps and parameters:*

1. *Zscore prior to averaging EEG evidence accumulation signal:
   previous:	with z-score:*

2. *And using a spatial filter 100 ms prior to the response instead of from the time of the response:*

3. *Calculating signal-RT correlation on log reaction times (dashed lines) compared to untransformed (solid lines) and using a fisher transform:*

4. *With this, and using the parameters fit from the simulation-based approach instead of the prepaid approach:
   previous:	new:*
 *(Note that the inverse leak is taken relative to the bound, as the bound parameter was more variable across participants when fit with the simulation-based approach)*
5. *Calculating the EEG motor accumulation signal using slopes calculated over a shorter time-window (26 ms) and using sample rather than time in milliseconds as the coefficient:
   Previous:	New:*

6. *Estimating the weights for the EEG motor accumulation signal from data further prior to the response (-250 ms instead of -150 ms):*

**R1C6**
[C] "estimatable" probably should be "estimable"

*Thank you, we have made the correction.*


***Reviewer #2:

Verdonck et al. have recently proposed a dual integration model of perceptual decisions that are reported with a motor action (the Leaky Integrating Threshold or LIT model). Rather than applying a decision threshold to the integrated sensory evidence, as in the case of a typical single-stage integration-to-threshold model like the drift diffusion model (DDM), the integrated evidence is fed into another (leaky) integrator, which ends up being compared to a decision threshold. The authors propose that the mechanism for implementing the speed-accuracy tradeoff is a change in the time constant of the second integration stage.

In this manuscript the authors provide further experimental evidence for the two-stage model based on human behavioral and EEG data from a dynamic face vs. car categorization task.

I find the possibility of a dual-integration model interesting and some of the provided evidence, the observation of spatially segregated EEG signals with an appropriate relationship with each other as well as with the timing of individual decisions, quite convincing. At the same time, I have some questions about some of the choices/statements made by the authors, as further detailed below.

*We thank the reviewer for their careful reading of the manuscript and helpful comments. We find their suggestions particularly useful for improving the quality of the manuscript and hope to have fully addressed their comments.*

**R2C1**
1) The estimation method for obtaining the model parameters (D*M) is based on RT distributions, but we never get to see any RT distributions, neither those of the data, nor those predicted by the considered models. Could you please show some comparisons?

*We thank the reviewer for the helpful suggestion to show the RT distributions and model fits, which we have added to Figure 2:*

*“Density of correct and incorrect (plotted downward from 0) responses in equisized bins of response times summarised across participants (markers; yellow for high sensory evidence, red for low; open markers show the speed condition) and prediction from models (based on simulation; DDM with boundary separation mechanism in dashed and LIT in solid lines; green lines show the speed condition).”* ***P6L165***

*In response to the comments below, and concerns from Reviewer 1 about the approachability of the manuscript for a wider audience (and to estimate more traditional model comparison statistics;* ***R1C4****), we have re-fit the models using a simulation-based approach. In this approach the non-decision component of the RT distribution is estimated by taking a truncated normal distribution (with two free parameters, the mean and variance), which allows for rightward skew in the resulting RT distributions while being more computationally efficient than a gamma distribution. For parsimony, and in line with the comments below (especially* ***R2C2****), we assumed the same non-decision time across speed and accuracy conditions in this fitting approach. The RT distribution plots are the result of parameters from this simulation-based fitting procedure.*

**R2C2**
2) One of the characteristic predictions of the LIT model is that a change in the integration time constant of the second stage leads to a change in the timing of the decision that would have to be captured by a change in the nondecision time in the case of a standard DDM. I find it therefore quite surprising that the authors allowed the nondecision time distribution to change with speed/accuracy (urgency condition) in all their model fits, which could potentially undo those kinds of timing changes. Furthermore, as far as I can tell, the resulting estimates of the nondecision time distributions are never reported. Could you please show these (or demonstrate that the LIT model still performs well, when the nondecision time distribution is not allowed to change with speed/accuracy)?

*We thank the reviewer for this suggestion, and have now fit the models keeping the non-decision time fixed across SAT conditions, using a simulation-based approach which may be more approachable for a wider audience. We have added details of the additional fitting procedure to the methods:*

*“In addition to the grid-based search, we obtained parameter estimates for each observer for DDM and LIT models without re-parameterisation by minimising the negative log-likelihood of the data using Bayesian Adaptive direct search (Acerbi & Ma, 2017 [81]). The negative log-likelihood was estimated over 10 quantiles of reaction times using a Monte Carlo simulation procedure with 10000 samples per stimulus condition (Heathcote et al., 2002 [82]). Both models had four parameters for drift rate (v; one for each stimulus condition) and two parameters for non-decision time (the mean and variance of a truncated gaussian), which was kept constant across SAT conditions. For the DDM, SAT conditions were modelled with two separate bound parameters, while for the LIT there was one bound parameter and two separate leak parameters. Thus, the DDM had a total of 8 parameters and the LIT, 9. Model comparison was therefore conducted using the Bayesian Information Criterion (BIC).”* ***P22L626***

*The LIT suggests that changes in the speed-accuracy trade-off are controlled by changes in the motor accumulator leak, which affects both the rate of evidence build-up and the temporal smoothing. Specifically, in the accuracy condition, there is less leak and so greater temporal smoothing, resulting in less noise in the motor evidence. This reduction in the noise would manifest as less variability in decision times, and so reduced variability in response times, assuming the same non-decision time distribution. This effective relationship between the leak and what is normally parameterised as non-decision time is described by Equation 4, when reparameterising the LIT with the DDM parameters. We now highlight this more explicitly:*

*“Of note, Eq 4 presupposes a change in the apparent non-decision time according to the leak (due to the temporal smoothing).”* ***P19L579***

*For the DDM to capture this reduced variability in the accuracy condition, a change in non-decision time would be required. We therefore allowed the non-decision time to change according to the SAT condition so that the DDM would not be handicapped in this way. In other words, if we had fixed the non-decision time across SAT conditions, the DDM would not be able to capture changes in non-decision time across SAT conditions while the LIT effectively would. We have clarified this in the manuscript:*

*“The non-decision distribution is also allowed to differ between urgency conditions: as this is implied by the LIT (Eq 4), fixing non-decision time across conditions would handicap the other models and make this model comparison unfair.”* ***P21L610***

*However, we also appreciate that the changes in effective non-decision time are not normally substantial enough to merit additional parameters. While fitting the models with the simulation-based approach we compared models assuming the same non-decision time distributions or allowing the parameters of the non-decision time distributions to vary across SAT conditions. We indeed found that the additional parameters were not parsimonious in terms of BIC for the LIT (on average BIC increased by 3.97 when including the additional parameters). However, the DDM did benefit from the additional parameters in terms of BIC (decreasing BIC by 3.02 on average). In line with what we suggest above, the mean fitted non-decision time for the LIT varied across SAT conditions much less than for the DDM (40 ms vs 79 ms less in the accuracy compared to the speed condition) and the relative improvement in negative log-likelihood was smaller for the LIT than the DDM (3% vs 5.3%). The suggests that fixing non-decision time across SAT conditions is indeed a handicap for the DDM and therefore we have left the parameter free to vary across conditions in the reparameterised version of the models.*

**R2C3**
3) Related to this topic, the authors cite a few studies, which have reported that the nondecision time seemed to change with speed/accuracy when applying a DDM to behavioral data. This, however, is not true for all studies. Palmer et al. (2005) is a good example, where decision times were changed over a wide range based on speed/accuracy instructions, but a DDM was able to capture the behavior quite well with only a change in the decision bound and no systematic change in the nondecision time. It should at least be mentioned that there are studies that did not find a change in the nondecision time with speed/accuracy manipulations.

*We agree with the reviewer that non-decision time is not the most important parameter for capturing changes in reaction time across speed-accuracy trade-off conditions. In many situations the most parsimonious parameterisation is to fix non-decision time across SAT conditions, as we found in the analysis presented above. This doesn’t mean there are no changes in non-decision time.*

*Palmer et al. (2005) report: “Third, in the initial 3-parameter analysis of this experiment, the residual time parameter varied with speed stress. Specifically, increasing the target time decreased the residual time estimate.”*

*And: “In sum, adding parameter variability provides a more complete description of the data, albeit at the price of greater complexity.”*

*With the LIT, these small changes in non-decision time parameters can be captured by the very parameter that controls the speed-accuracy trade-off: the LIT provides a more complete description of the data without the price of greater complexity. Non-decision time has traditionally been treated as a nuisance variable and dealt with using nuisance parameters. The LIT suggests that some variability in what was considered non-decision time is actually the result of functional changes in decision processes. We hope to have clarified this in addressing* ***R2C2*** *above.*

*Given the current density of the manuscript, we have decided not to elaborate further on this discussion. We believe a more relevant discussion can be made in future research, where a robust exploration can be conducted with EMG data (for example, Dendauw, E., Evans, N. J., Logan, G. D., Gajdos, T., Haffen, E., Bennabi, D., & Servant, M. (2022 pre-print). A dual-stage dual-threshold evidence accumulation theory for decision-making, motor preparation, and motor execution. PsyArXiv* [*https://doi.org/10.31234/osf.io/dxsjh*](https://doi.org/10.31234/osf.io/dxsjh) *)*

**R2C4a**
4) In Fig. 3a the authors show accumulated evidence signals from a DDM locked to the decision threshold crossing. It is generally believed that the decision threshold crossing precedes the onset of the motor response and that part of the "nondecision" time that typically is fitted when fitting standard integration-to-threshold models is related to the preparation/execution of the motor response. Model signals that are time-locked to the decision threshold crossing should therefore not directly be compared with neural signals that are time-locked to the onset of the motor response.

*We agree that this applies to the standard DDM. The LIT models the accumulation of evidence in what we presume to be motor cortex, with the response triggered immediately after the evidence reaches the bound, travelling directly down the spinal cord to the finger. There would be relatively little non-decision time after threshold crossing, with the majority incurred from lower-level processes (e.g. in visual cortex). This is also supported by evidence from single cell recording suggesting motor accumulator neurons in M1 of non-human primates peak 140 to 125 ms prior to the behavioural response (Thura & Cisek 2014). We now note this explicitly in the manuscript:*

*“By directly modelling motor processes the LIT assumes the response (button press) is executed with only a small and systematic delay relative to the time the motor threshold is reached.”* ***P8L235***

**R2C4b**
In fact, models like the DDM do not specify how the accumulated evidence signal should behave after the decision threshold has been crossed, as it is not relevant for the outcome of the decision. It is, however, quite relevant when a comparison between neural activity immediately before the onset of the motor response and model predictions is made. The fact that accumulated evidence reached a fixed level at the time of the threshold crossing, regardless of the strength of the sensory evidence, does not mean that this would still have to be the case at the time of the onset of the motor response. It could, if integration stopped immediately at the time of the threshold crossing without any leak. It would not be expected to be the case, if integration continued after the threshold crossing. (And it is difficult to predict what exactly would happen, if activity started to leak away after the threshold crossing.) I.e., even if a threshold on the accumulated evidence (first and potentially only integration stage) were to terminate the decision, but the integration continued during a (roughly) fixed time interval between the threshold crossing and the onset of the motor action, a crossing of the accumulated evidence signals like the one shown in Fig. 3b would be possible. I would therefore like to see to what extent a single-stage integration model could still account for (some of) the features of the EEG signal that has been linked to accumulated evidence by the authors and that have been interpreted as evidence for the two-stage model (like the sign change of the black solid line in Fig. 3b prior to the onset of the motor response and the corresponding findings in the EEG signal in Fig. 4c and d).
That said, the change in the zero correlation lag with speed/accuracy could probably not easily be explained that way.

*This is a nuanced comment, for which we give more details below.*

*In short, the DDM predicts that the signal-RT correlation is unlikely to cross zero (only under specific circumstances), and is relatively independent of bound placement (it should not differ across speed and accuracy conditions). We’ve made this clearer in the manuscript:*

*“However, for the standard DDM the non-decision time is assumed to include additional time for the choice to be communicated to the motor cortex, after which the button press planned and executed. If evidence accumulation were to continue during this time with the same drift rate, and the time-course of evidence accumulation was locked to the time of the response, rather than the decision, the signal-RT correlation could become negative so long as there is not substantial variability in the non-decision time across trials. The timing of this zero-crossing is relative to the duration of non-decision time. Under the DDM framework the signal-RT correlation is dependent on the sensory evidence strength but not on the decision boundary separation, meaning that this model predicts no substantial difference in signal-RT correlations between speed and accuracy conditions.”* ***P8L236***

*We’ve also highlighted more explicitly the distinction between the predictions:*

*“Specifically, the LIT predicts that the primary evidence accumulation signal-RT correlation will cross zero, and the timing of this zero-crossing will be earlier in the accuracy condition than the speed condition (as it is dependent on the leak). In contrast, the DDM predicts signal-RT correlations that do not substantially differ between the speed and accuracy conditions.”* ***P8L247***

*We’ve also emphasised the distinction from our predictions:*

*“This pattern is inconsistent with the DDM which predicts no substantial difference in signal-RT correlations depending on boundary separation (the primary mechanism for SAT control under the DDM framework).”* ***P11L325***

*To fully clarify our response:*

*We agree that it is possible under a DDM framework there could be additional non-decision time after decision commitment in which the evidence continues to accumulate. As the reviewer points out, the behaviour of the accumulator after decision-commitment is a matter of contention, with some authors arguing for ‘absorbing’ or ‘reflexive bounds’ (where evidence can only decrease below the bound after it is initially reached, but not surpass it): Zhang, J., Bogacz, R., & Holmes, P. (2009). A comparison of bounded diffusion models for choice in time controlled tasks. Journal of mathematical psychology, 53(4), 231-241). With these other alternatives, the correlation between the accumulated evidence and reaction times would be close to zero after decision commitment (since the accumulation signal itself would have little variability).*

*Assuming continued accumulation, for a fixed time interval between the threshold crossing and the time of the button press, the correlation would reach 0 at the time of decision commitment (a fixed time prior to the button press) and remain at 0, never crossing. This is because at the time of the decision the accumulated evidence has the value of the bound, and then for a fixed duration after continues to accumulate at an average of the drift rate with added noise that is independent of the reaction time:*

*Here, yellow and red are simulations of the high evidence strength and cyan and blue low evidence strength stimuli in the speed and accuracy conditions respectively. The green and black lines show the correlation computed across evidence coherence levels for the speed and accuracy conditions respectively.*

*Of note, one can see that calculating the correlation across evidence strength conditions results in negative correlations (since after the bound, the low evidence strength accumulates less evidence relative to the high evidence strength), but still there would be no difference in the timing of the zero-crossing across speed/accuracy conditions.*

*Assuming continued accumulation, with some variability in the time interval between the threshold crossing and the time of the button press, the correlation can remain positive. This is because there is no time-point (relative to the response) at which the accumulated evidence is equal across decisions, and faster responses are made after steeper rate of evidence accumulation, with the majority of the evidence accumulated prior to the bound (aligned to the response, the steeper evidence accumulation traces are more likely to have a greater decrease one time-step before the response than the shallower rates of evidence accumulation):*

*We believe this makes a robust case for the double-integration framework and once again thank the reviewer for their suggestion.*

**R2C5**
5) Hanks et al. (2014) have previously reported invasive recordings from monkey LIP during a perceptual decision task with an urgency manipulation. Are the results that have been reported there (speed stress being mediated by an additive signal before the threshold crossing; not clear across monkeys whether this signal was constant or increasing over time, i.e., added to the output or to the input of an integrator) consistent with the LIT model? If so, would activity in LIP correspond to the first or the second integration stage?

*This is an interesting question that we hope will be addressed in future research. On the one hand, LIP has been implicated in the preparation of eye movements (the method of responding in that study), and so could correspond to the motor accumulator when the response is made with a saccade. Indeed, some studies (such as those pointed out by Dr Schall below) show a ramping that could be predicted by the second accumulator. On the other hand, brain regions may not contain fully homogenous sets of neurons. For example, in the Heitz and Schall (2012) paper kindly suggested by Dr Schall below, they separated neurons in the frontal eye field into ‘visually responsive’ and ‘movement neurons’. At the level of EEG we only have a noisy measure of large populations of neurons (more likely, mixes of large populations from various spatial regions of the brain). By formalising the distinction between primary and motor accumulation, we hope that the LIT will be helpful to those using techniques such as single-neuron recordings to clarify the nature of the neural responses, how these might differ more broadly across the brain, and help us better characterise the entanglement between making a decision and making a response based on that decision.*

References:

Hanks T, Kiani R, Shadlen MN (2014) A neural mechanism of speed-accuracy tradeoff in macaque area LIP. eLIFE 3:e02260

Palmer J, Huk AC, Shadlen MN (2005) The effect of stimulus strength on the speed and accuracy of a perceptual decision. Journal of Vision 5:376-404

*** Additional feedback from Reviewer 1 (regarding Reviewer 2 comments):
Thoughts on R2 pt 1:
The authors have done this in previous publications but it would be good to include here for completeness.

Thoughts on R2 pt 2:
Very useful point for the authors to address.

*We thoroughly agree and once again thank the reviewer for this comment, which we hope to have thoroughly addressed above.*

Thoughts on R2 pt 3:
A scholarly and reasonably thorough survey of this issue is merited in this manuscript. If the reviewer knows of other studies complementing Palmer 2005, it would be helpful to share them.

*We would also appreciate if the reviewer would share other studies complementing Palmer et al., 2005.*

Thoughts on R2 pt 4:
The duration of the ‘non-decision’ motor delay is an important detail, and the manuscript would be improved by its consideration. We should note that the duration of such an efferent “point of no return” depends on which body part is moving and in what way. The non-decision motor delay for saccades, for example, is about 10 ms, and that’s all because of the brainstem mechanisms generating the ocular rotation. In the present study, participants indicated their choice via a button press using the index finger of either their right or left hand. The relationship between “decision” and “movement” thresholds has been investigated recently by Mathieu Servant, e.g., (Servant et al JNeurosci 2015 "Using Covert Response Activation to Test Latent Assumptions of Formal Decision-Making Models in Humans"; Servant et al J Exp Psychol Gen 2021 "An integrated theory of deciding and acting").

*We agree that this is an exciting avenue of further research and especially look forward to further work using EMG and measures of motor effectors to better capture the onset of movement. We believe that Mathieu Servant and colleagues are also exploring how a double-integration framework can help explain the relation between decision and movement (Dendauw, E., Evans, N. J., Logan, G. D., Gajdos, T., Haffen, E., Bennabi, D., & Servant, M. (2022). A dual-stage dual-threshold evidence accumulation theory for decision-making, motor preparation, and motor execution. PsyArXiv* [*https://doi.org/10.31234/osf.io/dxsjh*](https://doi.org/10.31234/osf.io/dxsjh) *) and look forward to seeing how this research progresses.*

Thoughts on R2 pt 5:
As noted in my review, the Heitz & Schall (2012) report on neurophysiology of SAT in monkeys reports more happening than the Hanks et al. study. As we are now comparing these studies, we should note that whereas the Heitz study sampled neurons while monkeys alternated between short blocks of Fast and Accurate trials, the Hanks study sampled some neurons when speed was emphasized and other neurons when accuracy was emphasized. In their data, no neuron was sampled in both conditions. This limitation confounds confident interpretations. The authors should also be aware of the Thura & Cisek work on neurophysiology of SAT in a different task (i.e. Thura et al. J Neurosci 2014 "Context-dependent urgency influences speed-accuracy trade-offs in decision-making and movement execution")

*We thank Dr Schall for these comments, which have helped us to address reviewer 2’s points. We especially appreciate the nuanced knowledge of the literature, in particular, the neural recording studies, as highlighted above. We are excited to continue exploring the utility of the LIT, where Dr Schall’s comments have made it clear that this framework may also bring additional clarity to studies of decision-making in non-human primates and rodents. Of course, the work of Thura and Cisek has been an inspiration for the LIT, both at the experimental and theoretical levels, since it has highlighted the involvement of motor processes in decision-making and the need for flexible adjustment in decision processes over and above boundary adjustments.*
