# Supplementary material for: Secondary motor integration as a final arbiter in sensorimotor decision-making
Source: PLoS Biol. 2023 Jul 17;21(7):e3002200. doi: 10.1371/journal.pbio.3002200 (PMC10393169; doi:10.1371/journal.pbio.3002200)
Supplement: Attachment — Submitted filename: response_review2.docx [file pbio.3002200.s003.docx]

We outline below our point-by-point response to the additional comments from reviewer 1.

Reviewer remarks:

Reviewer #1, Jeffrey D Schall (note: reviewer 1 signed this review): The authors have responded to the comments, critiques, and suggestions very well. I have no further comments to make about the revised manuscript, which is improved empirically and conceptually. However, in the spirit of scientific inquiry, let me offer some reactions to the comments of the other referees as they relate to this manuscript and associated work. Let me preface these remarks by stating that I am *not* seeking more citations, just better understanding.

R2C4a, the referee wrote: "It is generally believed that the decision threshold crossing precedes the onset of the motor response and that part of the "nondecision" time that typically is fitted when fitting standard integration-to threshold models is related to the preparation/execution of the motor response. Model signals that are time locked to the decision threshold crossing should therefore not directly be compared with neural signals that are time-locked to the onset of the motor response." - This comment is based on some architectural suppositions that recent work has addressed. Principally, the neurons doing the accumulation of evidence in the Gated Accumulator Model of Purcell et al. are the presaccadic movement neurons that can also be described as "motor preparation". In fact, in other work we showed that the neurons in FEF doing evidence accumulation are also doing response preparation and inhibtion: Middlebrooks PG, Zandbelt BB, Logan GD, Palmeri TJ, Schall JD. (2020) Countermanding perceptual decision-making. iScience 2020 23(1):100777. Because this manuscript is advancing the literature in its identification of distinguishable stages of processing, it seems useful to think carefully about the propositions linking particular neurons with particular processes. For what it's worth, I've meditated on this problem in this recent review: Schall JD (2019) Accumulators, neurons, and response time. Trends in Neurosciences. 42(12):848-860.

We thank the reviewer for this additional remark, which we believe supports our initial response to reviewer 2’s comment. We have added the reference to Schall (2019) to the relevant part of the discussion:

“Overall, the LIT has important neurobiological implications as it highlights the need to differentiate between two inter-related but largely separate accumulation processes that are likely to take place in different brain networks…”

R2C5, in reply the authors wrote, "On the other hand, brain regions may not contain fully homogenous sets of neurons." - The heterogeneity of neurons in every cortical area has been established beyond dispute. We see it in FEF, e.g., Lowe Kaleb A. and Schall JD (2018) Functional categories of visuomotor neurons in macaque frontal eye field eNeuro 5(5): ENEURO.0131-18.2018. doi: 10.1523/ENEURO.0131-18.2018. It is also the case in LIP (e.g., Meister ML, Hennig JA, Huk AC. Signal multiplexing and single-neuron computations in lateral intraparietal area during decision-making. J Neurosci. 2013 Feb 6;33(6):2254-67), even though many authors do not incorporate such differences into their theoretical outlooks.

We thank the reviewer for this additional remark, which we have now made more explicit in the manuscript:

“Overall, the LIT has important neurobiological implications as it highlights the need to differentiate between two inter-related but largely separate accumulation processes that are likely to take place in different brain networks [70 (Schall,2019)] or even heterogenous neural populations [71 (Lowe et al., 2018)].”

Reviewer #2, Jochen Ditterich (note reviewer 2 signed this review): Thank you for your detailed response. All of my comments have been successfully addressed.

We thank the reviewer for their ongoing support of the manuscript and are glad they feel we have sufficiently addressed their previous comments.
